# From state commodification to local reproduction of vulnerability: ethnographic insights from a Risk Zone Urban Renewal Project in Turkey

**DOI:** 10.1111/disa.70024

**Published:** 2025-10-03

**Authors:** Cansu Civelek

**Affiliations:** ^1^ College of Fellows University of Tübingen Germany

**Keywords:** commodification of vulnerability, disasters, earthquake, reproduction of vulnerability, Turkey, urban governance, urban renewal

## Abstract

This paper explores how vulnerability is not only defined by the state but also actively reshaped through policy implementation and lived experience. Drawing on ethnographic research in Eskişehir, Turkey, I propose an analytical distinction between the ‘commodification of vulnerability’—framing risk in technoscientific and moral terms to justify intervention—and the ‘reproduction of vulnerability’—capturing the emergent precarity produced by such action. Following the 2011 Van earthquakes, Turkey's central government advanced disaster prevention as a national imperative. In 2012, Law No. 6306 was enacted to facilitate large‐scale urban transformations in the name of risk prevention. Eskişehir's Metropolitan Municipality quickly adopted the ‘Renewal Law’, initiating the Risk Zone Urban Renewal Project with the stated aim of protecting lives and property. While official definitions of vulnerability centred on the structural durability of the built environment, residents of the designated area have encountered new and unanticipated vulnerabilities, ranging from housing insecurity and prolonged legal limbo to socioeconomic instability and emotional distress.

## 1 INTRODUCTION

*Eskişehir's Risk Zone is located in a second‐degree seismic zone. The high number of vulnerable buildings, along with significant soil liquefaction, increases the risk of disasters in the event of an earthquake* (Eskişehir Metropolitan Municipality and İTÜ, 2017, p. 16).

*We know nothing about the project. We keep waiting and the municipality wants us to believe that they will save us. But we are already vulnerable because of the project* (female resident of the Risk Zone, author's field notes, 11 September 2018).

The first quote reflects the official definition of vulnerability articulated by Eskişehir Metropolitan Municipality and Istanbul Technical University (İTÜ)^1^ in Turkey, which are seeking to implement an urban renewal project in anticipation of future disasters. However, the local population experiences and interprets vulnerability in more complex and multifaceted ways—reconfiguring its meaning through lived realities, as I explore throughout this paper.

Eskişehir, located in northwest Turkey, has undergone numerous urban transformation projects, particularly since 1999 when the mayor of the metropolitan municipality was elected from the centre‐left Republican People's Party (CHP), with promises of modernisation and Europeanisation (Civelek, 2024). The municipality has utilised a variety of narratives to justify urban transformation, including beautification, economic revitalisation, the boosting of tourism, and serving the public interest (Civelek, 2024). Amid these claims, in 2013, the rhetoric of ‘disaster risk prevention’ entered the municipality's agenda, especially following the introduction of Law No. 6306, *The Law on the Transformation of Zones at Risk of Disaster*, by the ruling Justice and Development Party (AKP) in the aftermath of 2011 Van earthquakes.

In 2013, Eskişehir Metropolitan Municipality invoked Law No. 6306 to initiate the Risk Zone Urban Renewal Project, aiming to advance a long‐standing redevelopment agenda (Civelek, 2023). While its rhetoric emphasised the physical vulnerability of the built environment, the local population's understanding of vulnerability has been shaped by a broader set of concerns, including economic insecurity, social displacement, and the uncertainty produced by prolonged policy ambiguity. Consequently, this research investigates how state‐defined notions of vulnerability diverge from residents' lived experiences and how new vulnerabilities are reconfigured as a result of anticipatory disaster governance. Harnessing the power of ethnographic study, this paper examines how local knowledge, everyday practices, and social relations unravel/elucidate forms of precarity obscured by official definitions and quantitative indicators.

To disentangle analytically the multiple operations of disaster governance, I propose a conceptual distinction between the *commodification* and the *reproduction* of vulnerability (see Table 1). The commodification of vulnerability refers to the state's technocratic and anticipatory framing of risk—typically constructed using seismic data, structural surveys, and generalised policy language—that makes certain populations and territories targets for intervention. Within this logic, vulnerability becomes a measurable and depoliticised condition that justifies market‐driven solutions, such as large‐scale urban renewal projects. This process not only flattens complex lived realities but enables vulnerability to circulate as a form of value—something to be acted upon, acquired, and even capitalised through legal and financial mechanisms, such as property expropriation and zoning redesignation. In contrast, the reproduction of vulnerability refers to the new or intensified forms of precarity and harm that emerge from these very interventions. They include legal uncertainty, displacement, psychological stress, erosion of social networks, and the disempowerment of communities, rendered passive in the face of bureaucratic authority. In this sense, vulnerability is not only framed and acted upon, but also it is actively (re)produced through the material, emotional, and spatial consequences of so‐called disaster prevention. This distinction helps to illuminate how vulnerability, far from being a fixed or pre‐existing trait, is simultaneously a political construction and a lived condition shaped by intersecting regimes of power.

**TABLE 1:** The commodification and the reproduction of vulnerability.

Concept	Definition	Operational form	Primary actor	Temporal focus
*Commodification of vulnerability*	Framing vulnerability as a measurable, technical condition that justifies state or market interventions	Legal classification (such as ‘risky areas’), planning documents, expropriation	State, technocrats, investors	Anticipation (pre disaster)
*Reproduction of vulnerability*	The emergence of new forms of harm and precarity caused by preventive interventions	Displacement, uncertainty, exclusion from decision‐making, loss of rights	Affected communities	Ongoing, chronic, open‐ended

**Source:** author.

Methodologically, this research is grounded in long‐term ethnographic fieldwork conducted in Eskişehir, Turkey, with a focus on the Risk Zone Urban Renewal Project. Adopting a slow‐research approach (starting in 2013 and still ongoing), I engaged in sustained participant observation, in‐depth interviews, informal conversations, and document analysis to trace how vulnerability is defined, negotiated, and contested at multiple levels. I conducted hundreds of semi‐structured interviews and informal conversations with residents with diverse socioeconomic, ethnic, and generational backgrounds, many of whom have lived in the designated risk area for decades. Sampling was primarily purposive and snowball‐based, guided by the goal of capturing a broad range of perspectives, including those of homeowners, tenants, shopkeepers, women, youth, and elderly residents.

In addition to community voices, I carried out more than 100 formal interviews and semi‐structured interviews with key institutional actors, including municipal officers, urban planners, and members of professional organisations such as the Eskişehir branch of the Chamber of Geological Engineers, which has been publicly critical of the Project. Between June and September 2016, I also conducted extended observations at the Risk Zone Communication Centre—an official municipal space where residents met with officials to discuss the Project's details. These moments offered unique insights into the affective, procedural, and often ambiguous exchanges between the state and citizens.

I also visited the offices of locally elected representatives in all eight neighbourhoods included in the Risk Zone Urban Renewal Project, where I held interviews with them and informal conversations with local residents. In addition, I was invited to neighbourhood meetings, workshops, and public forums to observe how the Project was communicated and contested in collective settings. This allowed me to attend to what was said in interviews, as well as what was performed, withheld, or emotionally expressed in everyday encounters. The ethnographic depth of this study is not merely to document events, but also to illuminate the affective and relational dimensions of disaster governance, which are often overlooked in policy documents and cannot be fully captured through quantitative indicators.

I begin the discussion by situating Turkey's disaster capitalism agenda within critical disaster studies. I then trace the genealogy of vulnerability studies to position my contribution with respect to the existing literature. After outlining the political economy of urbanisation under the AKP's rule, I examine the case of Eskişehir from the viewpoints of the commodification and the reproduction of vulnerability.

## 2 DISASTER CAPITALISM AND TURKEY'S URBANISATION AGENDA

Since the end of the Second World War, anthropologists and sociologists have analysed the social roots and implications of disasters, using them as lenses to understand broader societal elements and social realities (Simpson, 2013; Matthewman, 2015, p. 11; Schuller and Maldonado, 2016, p. 62). While various disciplines, including geology, geography, psychology, and economics, have investigated hazards and risks, recent decades have seen the development of a critical perspective in the field of anthropological and sociological disaster research. This approach seeks to move beyond objectivist, technocratic, quantitative, and individualistic analyses of disasters and vulnerability, emphasising instead the social construction of risk.

Comprehending disasters as socially constructed processes stands in contrast to psychological studies that adopt a methodological individualistic perspective, focusing narrowly on personal vulnerabilities without considering wider social, cultural, and economic factors. Disasters are not simply material events that alter the built environment and physical space (García‐Acosta, 2002), and they cannot be fully understood based on numerical calculations or individual trauma alone; rather, they are social events emerging from interlinking—and frequently conflicting—processes that transcend traditional boundaries of time and space (Oliver‐Smith, 2002; Button and Schuller, 2016). Risk creation, allocation, and management, as well as disaster policies and vulnerabilities, have deep historical, social, political, and economic roots within society (Tierney, 1999; Oliver‐Smith, 2002; Matthewman, 2015; Barrios, 2016; Button and Eldridge, 2016; Faas, 2016).

In contrast to traditional techno‐scientific disaster research, critical anthropological and sociological studies place disasters within a broader processual context, calling for interdisciplinary inquiries that combine social, economic, and political investigations and historical, archaeological, demographic, and medical perspectives (García‐Acosta, 2002; Oliver‐Smith and Hoffman, 2002, p. 6). Developing the work of Hewitt (1983), Oliver‐Smith (2009, p. 120) underlines that this shifts the research spotlight away from a disaster itself and towards the ongoing societal relations that prefigure the event.

Disasters are social phenomena that exist within a complex web of interwoven and often conflicting processes of social construction (García‐Acosta, 2002; Oliver‐Smith, 2002). Their impacts are reflected in socioeconomic policies as well as in production and distribution systems (García‐Acosta, 2002). Moreover, public concern about risk is shaped by the demands of prevailing ideologies, power structures, and economic programmes. More than 40 years ago, Douglas and Wildavsky (1983) identified the criteria with which societies determine what constitutes danger and risk, analysing why certain threats are prioritised over others within specific temporal contexts. Globally, public preoccupations with risk are influenced by socio‐temporal dynamics, underscoring that the creation, definition, and representation of risk are inextricably linked to the reproduction of power and ideology.

Disasters open up avenues for social and economic transformations, while ‘evidence‐based’ practices and ‘future estimations’ mediate the definitions and perceptions of risk, which are crucial components of establishing hegemony (Oliver‐Smith, 2002; Button and Schuller, 2016). Political and economic forces not only create and organise but also anticipate and profit from disasters, with recovery plans increasingly implemented according to neoliberal urban principles across both urban and rural landscapes. The reconstruction efforts following devastating disasters, such as Hurricane Mitch in Honduras (1998), Hurricane Katrina in New Orleans, Louisiana, United States (2005), and the earthquake in Haiti (2010) (Klein, 2007; Collins and Jimenez, 2012; Schuller and Maldonado, 2016), often lead to dispossession, eviction, loss of social networks, and social fragmentation. This disaster‐induced urban transformation finds a parallel in Turkey, where disaster narratives and disaster capitalism have been strategically instrumentalised to legitimise large‐scale urban renewal projects, particularly under the AKP government. I return to this case after outlining some key debates on vulnerability, especially within anthropological and critical disaster scholarship.

## 3 GENEALOGIES AND CRITIQUES OF THE CONCEPT OF VULNERABILITY

The concept of *vulnerability*—now central to disaster studies and humanitarian governance—emerged not in a vacuum but as part of a longer history of Western attempts to categorise and manage global inequality. As Bankoff (2001) argues, vulnerability is the latest in a lineage of discursive technologies that map certain populations and geographies as inherently deficient, dangerous, or exposed. Earlier concepts such as *tropicality*, *underdevelopment*, and *backwardness* played foundational roles in shaping a geography of moral and material inferiority. Tropicality, for instance, was a colonial construct that imagined equatorial regions not only as climatically unstable, but also as spaces of bodily degeneration and moral laxity—requiring external discipline and improvement. Similarly, Cold War‐era notions of underdevelopment and backwardness located responsibility for poverty and risk within the cultural or environmental limitations of the Global South, legitimising interventionist logics.

Bankoff (2001) asserts that the rise of *vulnerability* in the late twentieth century must be understood as part of this epistemic lineage. Although seemingly more neutral and technocratic, vulnerability continues to function as a moral–political category that reproduces older hierarchies. It casts entire populations and regions—often in the Global South—as permanently at risk, reinforcing paternalistic narratives that justify external control. In this framing, disasters are not the result of specific historical or political decisions but instead are treated as naturalised expressions of preexisting frailty. Such a perspective deflects attention away from structural drivers of risk, such as dispossession, state neglect, or uneven development, and reframes them as problems of spatial exposure and inadequate adaptation.

Building on this critique, Marino and Faas (2020) contend that vulnerability has become an increasingly hollow signifier in disaster and climate governance. While its usage has proliferated, it is frequently invoked without clarity about who or what is vulnerable, and why. This conceptual vagueness allows vulnerability to operate as a flexible tool of governance—capable of justifying intervention without addressing the deeper dynamics of power, responsibility, and resistance. Moreover, it risks reifying vulnerability as a trait of people or places, rather than as a product of political–economic processes. Like its predecessors, vulnerability uses a language that can obscure more than it reveals.

Rather than abandoning the concept entirely, Marino and Faas (2020) call for a critical re‐engagement that centres the structural, relational, and historical conditions under which vulnerability is produced. This includes paying attention to how the term is operationalised in legal and policy regimes, how it is used to justify particular spatial interventions, and how affected communities contest or rework its meaning. In this way, vulnerability is not only a condition to be measured but also is a contested terrain where competing claims over risk, value, and justice are negotiated.

This theoretical reframing resonates with the empirical concerns of this paper. Calculating the impact of disasters and the vulnerability of populations cannot be attributed solely to accidental geophysical features. Instead, vulnerabilities are shaped by the structural and historical conditions of inequality, accountability, transparency, and systems of production, exploitation, and social justice (Oliver‐Smith, 2002; Tierney, 2014; Matthewman, 2015; Barrios, 2016; Faas, 2016). As critical disaster research has shown, disasters are experienced differently by various groups and individuals, influenced by their political, economic, and social standing. Lower class, minority, and politically less organised communities tend to face greater exposure to the negative outcomes of disasters (Tierney, 1999; Oliver‐Smith and Hoffman, 2002). These disparities are rooted in the uneven distribution of housing and infrastructure, as well as rights, legal protection, and access to social services.

Thus, the everyday conditions of social inequality are not just amplified during disasters but constitute the very terrain of vulnerability itself. As Okoko (2024) emphasises, vulnerability manifests in the chronic precarity of daily life and becomes acutely visible in moments of crisis. In this sense, vulnerability is not merely a descriptor of risk but a reflection of systemic social violence. It is not only ‘socially constructed’, but once in place, it generates further consequences—both institutional and material—that reshape the lives of those subject to it.

Building on these theoretical contributions, I extend the debates by examining how vulnerability is mobilised and contested in Turkey. I go on to develop my contribution in detail when discussing the Risk Zone Urban Renewal Project in Eskişehir.

## 4 URBAN RENEWAL, THE POLITICAL ECONOMY OF AKP‐ERA URBANISATION, AND LEGAL INTERVENTIONS

Turkey's urbanisation process has undergone significant changes since 2002, when the AKP, under the leadership of Recep Tayyip Erdoğan, came to power. In response to the financial and economic crisis of 2001–02, the new government stimulated the construction sector (Candan and Kolluoğlu, 2008; Türkün, 2011; Saraçoğlu and Demirtaş‐Milz, 2014). Consequently, the number of urban renewal projects increased drastically, generating immense profits for both the public and private sectors; however, they also led to various forms of dispossession, eviction, and exclusion, affecting especially the urban poor and slum settlements (Şengül, 2009; Civelek, 2019).

Since 2002, the state has implemented several policy interventions encouraging the reproduction of urban built environments. While these large‐scale urban projects were being accelerated across Turkey, the AKP government continuously (re)invented new rationales for urban renewal policies, including the promotion of welfare through homeownership, long‐term savings for homeowners, and, more recently, vulnerability reduction projects aimed at mitigating the impacts of anticipated future disasters.

In the aftermath of the 2011 Van earthquakes, the Turkish government also utilised disasters and speculation about forthcoming catastrophes to emphasise the urgency of developing ‘safe and secure’ towns. Under the strategy, public institutions were granted the authority to undertake urban renewal initiatives in the name of disaster prevention. By recurrently referencing earthquakes and their victims, the government engaged in what Ophir (2010) describes as the ‘politics of catastrophization’, spreading threats of fear and anxiety while simultaneously framing the moral obligation to construct safe cities to avert imagined future disasters. This approach created fertile ground for alarming the public about the necessity of urban renewal, reducing the rationale for such projects to mere technical interventions. The tactics effectively obscure the need to address structural inequalities, underlying social contexts, and the neoliberal logic driving these initiatives (Saraçoğlu and Demirtaş‐Milz, 2014; Parson, 2016). During a meeting in Istanbul in 2012, the then Minister of Environment and Urbanism, Erdoğan Bayraktar, stated that there were 19 million dwellings in Turkey, 40 per cent of which required renewal. In May 2012, Law No. 6306— *The Law on the Transformation of Zones at Risk of Disaster*—was enacted, despite widespread opposition and criticism, including strong dissent from the centre‐left CHP in the Grand National Assembly. The principles of ‘disaster capitalism’ (Klein, 2007; Collins and Jimenez, 2012; Schuller and Maldonado, 2016) enabled the Turkish government to designate both public and private lands as risk zones, thereby facilitating their renewal.

Law No. 6306 functions not only as a legal tool for post‐disaster recovery but also as a core component of an anticipatory governance regime that restructures urban space in advance of catastrophe. Kayaalp (2025) argues that the legislation facilitates a form of legal engineering in which the state mobilises the possibility of disaster to justify pre‐emptively large‐scale spatial interventions. This anticipatory logic grants expansive discretionary power to state institutions such as the Ministry of Environment, Urbanisation and Climate Change and TOKİ (Mass Housing Administration of the Republic of Turkey), allowing them to override other legal protections such as Law No. 3621 (*Coastal Law*, 1990) or Law No. 5366 (*Law on Renovating, Conserving, and Actively Using Dilapidated Historical and Cultural Immovable Assets*, 2005) (Kızıldere, 2025). As a result, disaster governance is not primarily reactive but speculative—shaped by imagined futures that rationalise present‐day dispossession (Güney, 2024).

As Kızıldere (2025) demonstrates, the designation of ‘risky’ and ‘reserve’ areas is often decoupled from actual seismic risk, targeting neighbourhoods not for their physical precarity but for their rent‐gap potential. This instrumentalisation of vulnerability allows urban policy to function as a form of accumulation by dispossession. Risk is commodified and used to justify the seizure and redevelopment of land in high‐value districts (Güney, 2024). Hence, the law reconfigures vulnerability as a technocratic category aligned with investment interests, while masking the political and classed dimensions of exposure and displacement. Similarly, Kayaalp (2025) and Civelek (2025) frame this legal restructuring as a mode of anticipatory governance, wherein the state pre‐emptively mobilises earthquake risk to justify interventions that benefit political and commercial interests rather than communities at risk.

Furthermore, recent amendments to Law No. 6306 exacerbate the dispossession of residents. Kızıldere (2025) details how the redefinition of ‘reserve areas’ allows for the seizure of existing residential plots of land even when they are not at risk. Moreover, the shift from requiring a two‐thirds majority to a simple majority for redevelopment (Art. 6/1) further erodes participatory decision‐making. Residents who cannot pay for redevelopment may lose their home and, ultimately, their right to pass it on to an heir.

It is within this wider context of anticipatory urbanism, legal centralisation, and disaster capitalism that the Risk Zone Urban Renewal Project in Eskişehir must be understood. I turn to my case study now to examine how these dynamics have played out on the ground—how vulnerability is officially defined, how it is experienced and reconfigured by residents, and what these tensions reveal about the politics of urban renewal in contemporary Turkey.

## 5 ESKIŞEHIR'S RISK ZONE URBAN RENEWAL PROJECT

Significantly, the centrally produced discourses on risk and vulnerability have been reproduced by local governments, regardless of party differences, with the law being swiftly applied to various urban renewal projects. Despite CHP opposition, Eskişehir's CHP municipality quickly adopted Law No. 6306 and declared the Risk Zone Urban Renewal Project within a year. The context of earthquakes, collective shock, and the commodification of disaster discourse provided authorities with market opportunities to promote radical urban plans and policies.

In Eskişehir, the metropolitan municipality used the law to advance, in 2013, an ambitious urban renewal initiative known as the Risk Zone Urban Renewal Project. This was, crucially, the municipality's third attempt to renew the same area, each time invoking different rhetoric and legal frameworks.^2^ The previous two attempts were thwarted by local opposition and court rulings. This time, though, the municipality seized upon the emergency rhetoric and new legal framework introduced by the AKP government to push forward its long‐standing ambitions for accumulation (Civelek, 2023). The strength of Law No. 6306 was also harnessed to suppress local resistance. Consequently, disaster capitalism (Klein, 2007) and the emergency call were operationalised to drive urban policies that fulfilled the economic and political ambitions of a local government that had previously been unable to achieve them.

The Project targeted a 56‐hectare riverfront area in the city centre, home to approximately 15,000 residents and workers. What is particularly relevant for this paper is the heterogeneity of the building stock and its varied uses—residential, commercial, and mixed‐use—and the diverse population. As I will discuss, this heterogeneity positioned people differently in relation to the Project, leading to different forms of vulnerability.

**FIGURE 1:**
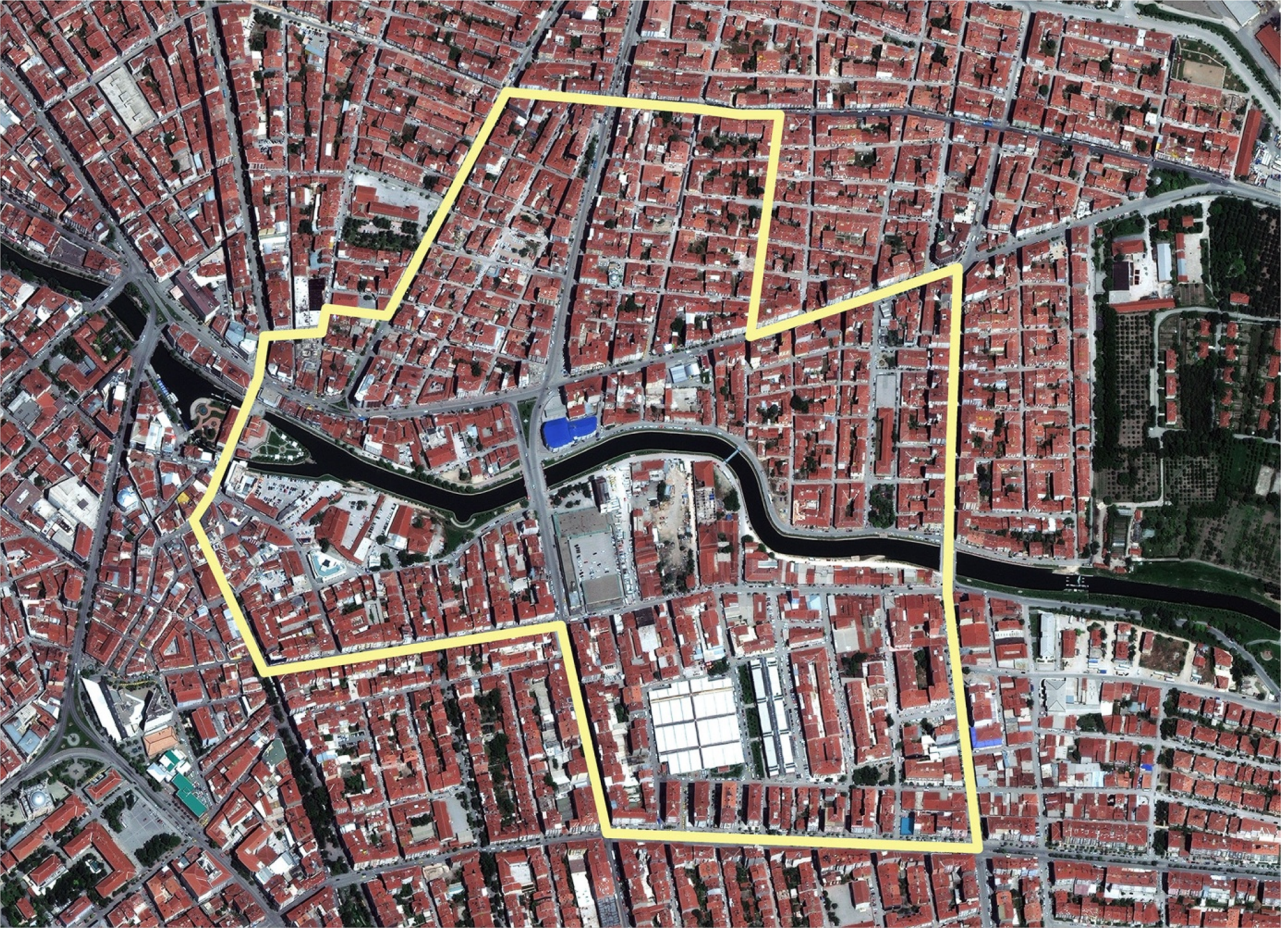
The borders of the Risk Zone, 21 June 2016. **Source:** Eskişehir Metropolitan Municipality, reproduced with permission.

**FIGURE 2:**
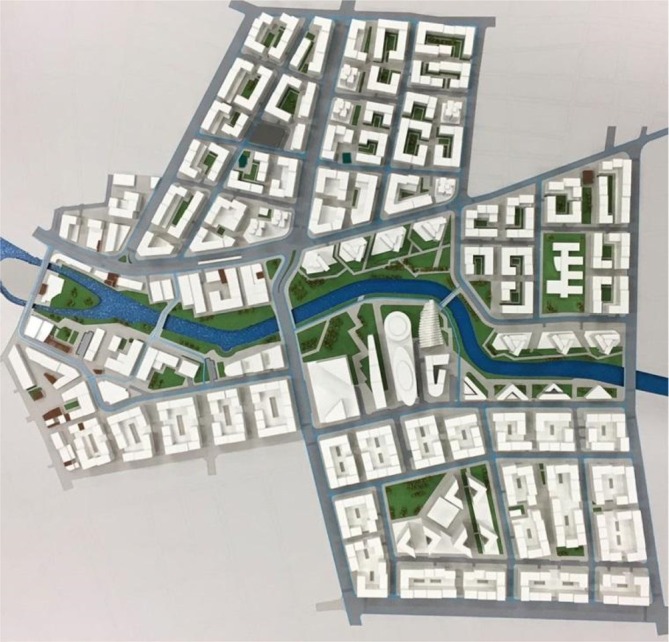
The plan for the Risk Zone Urban Renewal Project, 21 June 2016. **Source:** Eskişehir Metropolitan Municipality, reproduced with permission.

Discourses surrounding future earthquake victims were followed by the municipality's techno‐moral claims of responsibility to save lives. The Risk Zone was officially approved by the Council of Ministers on 17 April 2013 and announced in the *Official Gazette* on 17 May 2013 (see Figures 1 and 2). Shortly afterwards, on 30 May 2013, the then Ministry of Urbanism and Environment authorised Eskişehir Metropolitan Municipality to conduct research and draft plans. By late 2013, the municipality delegated urban planning responsibilities to a team from İTÜ, which spent nearly three years (from 2014–17) conducting research on housing and building values, as well as the geological structure of the area, to develop the project plans. Between 2017 and 2019, several revisions were made in response to requests from the Ministry of Urbanism and Environment. In May 2019, however, the Council of State annulled the project following a court case filed by an individual contesting the Risk Zone.

Drawing on critical disaster studies and debates that conceptualise vulnerability as historically and socially constructed, this paper extends the literature by underscoring how populations experience vulnerability not merely as exposure to hazards, but also as dislocation, uncertainty, and precarity produced through disaster governance itself. It theorises vulnerability as a dynamic outcome of anticipatory governance—shaped by legal, political, and infrastructural processes that actively redistribute harm even before disasters occur.

This paper seeks, therefore, to move beyond the prevailing emphasis in disaster research on the aftermath of catastrophic events by shifting the analytical lens towards the reconfigurations of vulnerability that happen in anticipation of disaster events. Rather than treating vulnerability as a static condition revealed by a hazard, I conceptualise it as a dynamic and unevenly distributed product of legal, economic, and political systems. I analyse how state‐led interventions—particularly those framed as disaster prevention—actively produce new forms of precarity through bureaucratic ambiguity, infrastructural neglect, and opaque implementation processes. In this context, vulnerability becomes both a discursive and material outcome of anticipatory governance, in which future risks are invoked to justify present‐day transformations of space and social life.

Additionally, this research expands the concept of vulnerability beyond physical exposure to encompass its psychological, mental, social, and economic dimensions. In the Turkish setting, where disaster preparedness has been institutionalised through Law No. 6306 and similar frameworks, official definitions of vulnerability remain narrowly technocratic, often limited to assessments of the structural integrity of buildings. By contrast, I show how communities experience vulnerability through dislocation, legal uncertainty, emotional exhaustion, and a profound loss of control over their futures. These aspects are rarely captured in state risk evaluations, yet they influence how people live with and respond to the creeping temporality of disaster planning. As I assert, disaster prevention projects do not simply mitigate risk, but can also generate complex new exposures and deepen existing social fractures.

This study contributes to critical disaster scholarship by illustrating that vulnerability is not just revealed by crisis but manufactured through policy. It highlights the need for a more relational and process‐oriented understanding of vulnerability—one that accounts for historical injustice, spatial inequality, and the anticipatory operations of the state. Instead of assuming that disaster governance necessarily protects the public, I interrogate how it may redistribute harm in ways that reinforce hierarchies of value, citizenship, and dispossession. Ultimately, I argue for an approach to vulnerability that foregrounds lived experience, political agency, and the contested terrain of safety, risk, and belonging in the urban landscape.

Next, I examine the official configuration of vulnerability as defined by state actors. I then turn to the more complex and contested reconfigurations of vulnerability as experienced and articulated by affected communities.

## 6 COMMODIFICATION OF VULNERABILITY

As discussed, public knowledge of vulnerability is not neutral: it is shaped by holders and networks of power that determine what is recognised as risk and what forms of intervention are legitimised. Ferrarese (2016, p. 69) contends that the political dimensions of vulnerability become most visible in the processes through which it is defined. Eskişehir's Risk Zone Urban Renewal Project exemplifies this dynamic: its emphasis on vulnerability and risk was constructed by municipal authorities, yet reshaped and contested through the lived experiences and interpretations of local populations. This paper does not suggest that the Project was purely a rent‐seeking initiative, nor does it deny the existence of physical vulnerabilities in the building stock. It maintains, though, that these vulnerabilities were selectively framed and strategically deployed—what I refer to as the *commodification of vulnerability*.

**FIGURE 3:**
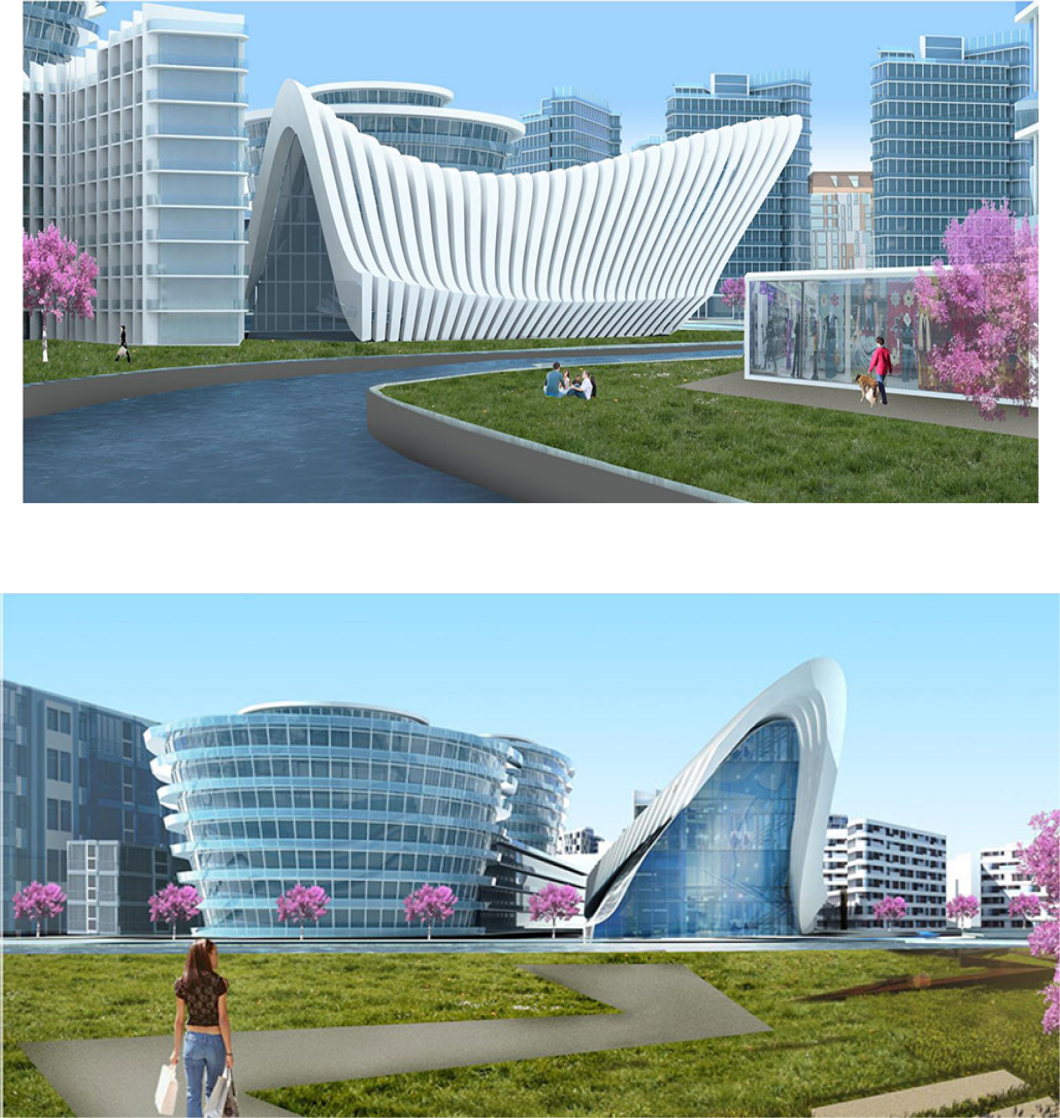
Visuals of the Risk Zone Urban Renewal Project, showing anticipated culture and leisure complexes along the river, 21 June 2016. **Source:** Eskişehir Metropolitan Municipality, reproduced with permission.

Via the commodification of vulnerability, the state reframed risk as a technical and moral imperative to justify urban transformation (see Figure 3). In this framing, vulnerability becomes an object of governance and intervention—abstracted from its social, historical, and economic contexts—and rendered actionable through policies such as Law No. 6306. While structural deficiencies may exist, the municipality's technocratic focus obscured a more complex reality: not all buildings were equally at risk, and not all residents experienced vulnerability in the same way. Meanwhile, new forms of vulnerability—legal uncertainty, housing insecurity, and psychological distress—were produced or exacerbated in the course of the Project's rollout and left unaddressed.

The production of ‘scientific facts’ plays a critical role in this commodification. As Alexander (2017) notes, the growing appeal of ‘evidence‐based practice’ among policymakers often masks the fact that evidence can be ambiguous, selectively mobilised, or disconnected from lived realities. In relation to the Risk Zone Urban Renewal Project, technical assessments were not simply diagnostic, but were also performative, legitimising pre‐defined solutions while marginalising alternative understandings of safety, risk, and vulnerability.

**FIGURE 4:**
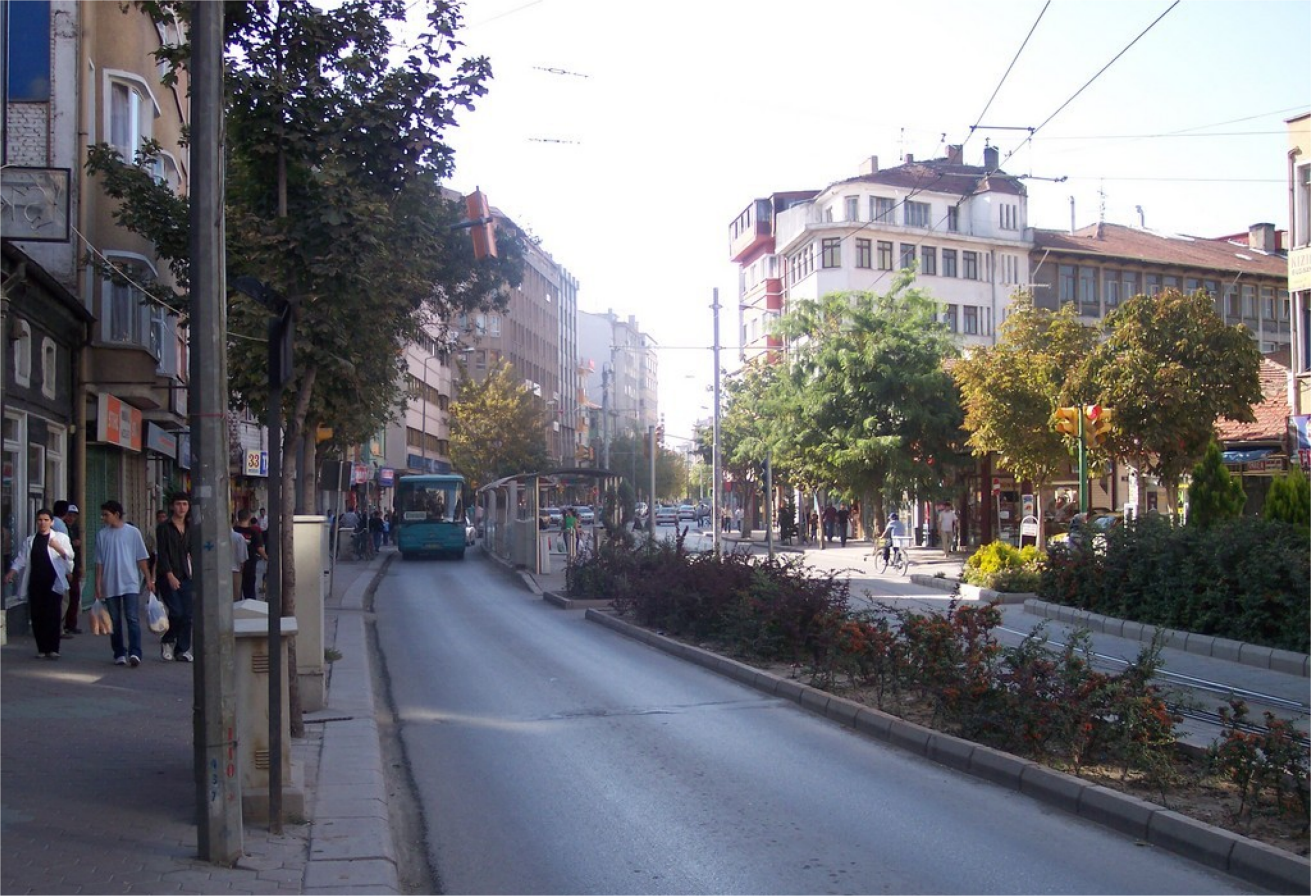
Street view of the commercial zone: Sivrihisar‐I Street, Mustafa Kemal Paşa neighbourhood in the Risk Zone, July 2015. **Source:** author.

**FIGURE 5:**
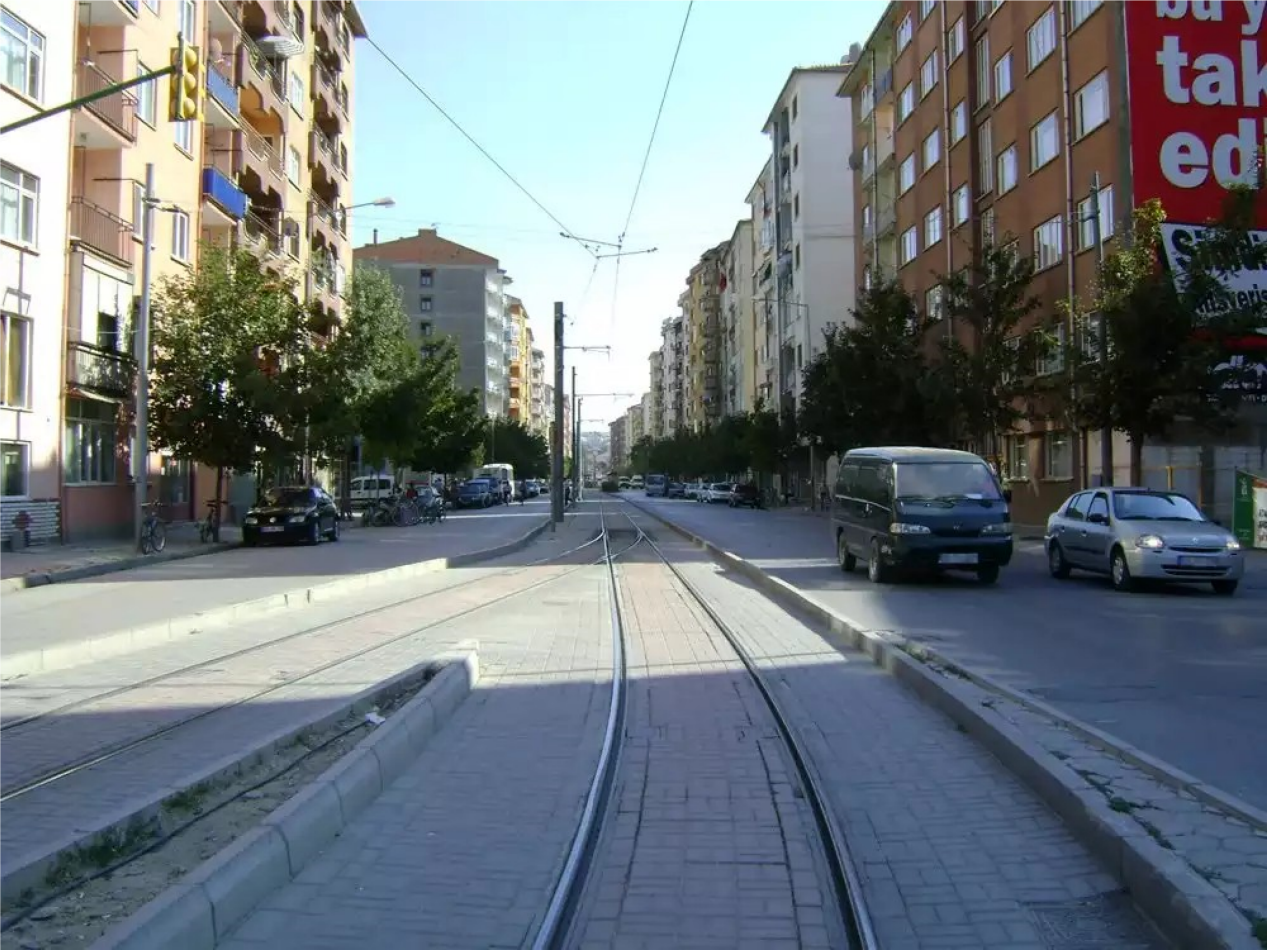
Housing and commercial zone: Hasan Polatkan Street, Mamure neighbourhood in the Risk Zone, July 2015. **Source:** author.

As noted earlier, the Project was Eskişehir Metropolitan Municipality's third renewal initiative for the same area (see Figures 4 and 5). Previous attempts were challenged in court, leading the municipality to adopt Law No. 6306, invoking claims of emergency and disaster risk prevention (Civelek, 2023). Scientific language and evidence‐based practices played a vital part when it defined risks and proposed ‘necessary solutions’. Yet, as Alexander (2017) argues, some of these diverged from people's realities, as will be discussed shortly. A report on the Project describes the risk of disaster in the event of an earthquake as follows:

*The most critical weakness of the zone is the soil liquefaction and vulnerable building stock. The greatest threat posed by this zone is its classification in a second‐degree seismic zone, which geologically necessitates risk prevention measures through this urban renewal project* (Eskişehir Metropolitan Municipality and İTÜ, 2017, p. 16).

The discourse surrounding risk and vulnerability has been underlined during public speeches, interviews, and press conferences. I attended the ceremony on 20 June 2016 to present the İTÜ's plans to the public, where the mayor of the metropolitan municipality delivered a speech invoking scientific evidence and underscored Eskişehir's obligations to address earthquake risks:

*After the 1999 earthquake, scientists emphasised two key points: first, we must learn to coexist with earthquake risks; and second, earthquakes do not kill—buildings do. Since then, I have been concerned about how we, as a responsible municipality, could manage earthquake‐disaster risks. Since I took office in 1999, we have been on a mission to combat earthquake risks. The central government's initiation of the law regarding the transformation of risk zones [Law No. 6306] has come to our aid, allowing us to begin work on a project for the shores of Porsuk* (author's field notes).

As Bankoff (2001, p. 27) asserts, the moral obligation to ‘save’ vulnerable populations often makes regions safer for investment and tourism. This motivation is evident in the case of the Risk Zone Urban Renewal Project. For instance, during the ‘RE360 Big Gathering for the Real Estate Industry’ in Istanbul in December 2022, the mayor of Eskişehir highlighted the municipality's growing modernity and tourism, emphasising the convenience and profitability of the Project for investors. He encouraged attendees to ‘turn away from Istanbul and look at Eskişehir’, claiming that ‘the Eskişehir Risk Zone project is a historical investment opportunity’.

Simultaneously, the Project purported to respect the social and cultural needs of local residents and claimed to implement a participatory approach (see Figure 4). I have discussed elsewhere how participation has become a performative act for local governments, creating distinctions between deserving and undeserving subjects (Civelek, 2023). Although the Risk Zone Urban Renewal Project asserted that it would consider the needs of citizens, aiming to ‘mark the distinctiveness of the Project’ as the mayor and the project leader from İTÜ emphasised during the opening ceremony, the selective participation of only wealthy zone owners rendered urban plans and policies inaccessible by lower‐class populations. Furthermore, the prolonged planning and policymaking process between 2013 and 2020 generated additional layers of uncertainty.

The research and planning process also lacked collaboration with other institutions, urban experts, and non‐governmental organisations in the city. Members of various departments of both universities and local chamber organisations, such as the Chamber of Geological Engineers and the Chamber of Civil Engineers, expressed concerns about the absence of communication and collaboration. For example, one professor from Anadolu University, an urban scholar and expert, noted that his institution learned about the Project through newspapers and was surprised that İTÜ was tasked with urban planning while none of Eskişehir's universities were invited to participate in deliberations (author's field notes, 16 March 2016).

Ultimately, the research and planning process was neither as transparent nor as participatory as the Project's policymaking claims suggested. Although the municipality celebrated its collaboration with public institutions, these efforts were limited to a select few local chamber organisations. The non‐collaborative and secretive nature of the policymaking and planning process hindered the municipality's ability to assess the needs of a highly heterogeneous population and understand its diverse realities.

The issue of risk was another significant topic of debate. The then head of the Chamber of Geological Engineers in Eskişehir remarked that the municipality could have prioritised riskier zones that required more immediate action. A geology professor from Anadolu University added that the designated Risk Zone was not among the riskiest areas. He questioned why the municipality chose this particular area for renewal, stressing that the Project would yield substantial profits, given its central and highly valuable location (author's field notes, 18 March 2016).

## 7 RECONFIGURATIONS OF VULNERABILITY

How do vulnerability reduction projects or policies generate new forms of emotional, social, and economic instability? Conducting ethnographic research across several years of planning and policymaking in relation to this Risk Zone allowed me to observe how new vulnerabilities emerged and were reproduced over time. While official definitions often *commodify* vulnerability—transforming it into a category to be managed, measured, and acted upon by state and project authorities—the *reproduction* of vulnerability highlights how these interventions can inadvertently reinforce and multiply precarities within affected populations. Selective participation mechanisms and the techno‐moral politics embedded in disaster prevention practices contributed significantly to this reproduction of vulnerability, producing new forms of exclusion and instability, which are examined in detail below. Furthermore, the pronounced heterogeneity within the Risk Zone produced differential impacts on residents. In the following subsections, I analyse the multiple dimensions of vulnerability—emotional, psychological, social, and economic—that surfaced owing to the Risk Zone's diverse demographic composition, including variations in class, gender, age, and other socioeconomic characteristics, alongside the heterogeneous conditions of the building stock itself.

### 7.1 Forms of building stock and property relations: emergence of emotional, mental, and economic vulnerabilities

The declaration of the Risk Zone Urban Renewal Project marked a turning point in property relations within the designated area. Once the Risk Zone was approved, construction and demolition permits were halted, ongoing projects were suspended, and risk annotations were added to existing title deeds. This led to a gradual depreciation in property values, making it increasingly difficult to buy or sell properties. Halime, a property owner who lived elsewhere, only became aware of the Project when she attempted to sell her flat. Upon discovering the annotation on her title deed, she realised that she could not sell it, as housing loans were no longer available for annotated properties (interview, 28 August 2017). Yavuz found himself in a similar predicament after deciding to sell his flat in the Risk Zone:

*This area [the zone by the river] was one of the city's most valuable areas during the 70s, 80s, and 90s. It may have lost some of its popularity in recent years, but it is still the heart of the city. I regret not selling my flat earlier. Now, after declaring this a Risk Zone, the property value has become a joke. I can't even buy a small cottage with what I'd get from selling this place. We've been deprived here. They say property values will increase by 400 to 500 per cent, but even if that happens, I'm not sure I'll live to see it. I feel utterly deprived* (interview, 3 September 2017).

In addition to declining property values, the heterogeneity of the Risk Zone's building stock introduced various new vulnerabilities. The area contains a mixture of one‐storey shops, houses, and taller buildings, up to eight storeys. Some buildings are 60–70 years old, whereas others were constructed just before the declaration of the Project. As a result, the buildings vary widely in terms of condition and needs.

Owners of newer buildings, constructed according to earthquake safety regulations, felt particularly affected. The decision to demolish the entire building stock, regardless of condition, led to significant distrust and a sense of injustice. Residents expressed stress, anxiety, and a loss of peace. I visited Ekrem's flat, built in 2012. A married man in his sixties and a father of two, Ekrem had purchased his first property at the age of 55; however, the Project changed everything for him:

*We've led modest lives, saving up everything to buy this flat with our retirement money and bank credit. I initially didn't worry because our building is new and earthquake‐safe. But then we were told that all the buildings would be demolished. I've lost my peace ever since. This is unbelievable, because the same mayor who I voted for gave us building permits and now he is saying that the entire area is a Risk Zone and must be demolished. What is more stupid is that we must pay for a new apartment if we want to stay here. I'm still paying off my loan, and this has caused me panic attacks. Seriously, I'm losing my mind. What did I do to deserve this?* (interview, 12 August 2017).

Similar mental and psychological health complaints were shared by other residents of newly constructed buildings. Elif, a married woman in her fifties, faced difficulties in her marriage after the declaration of the Risk Zone:

*We've lived here since I was born, and I got married here. We're a well‐known Tatar family in this neighbourhood. I could have moved to a different area, like Batıkent [a developing, middle‐class neighbourhood]*, *but I wanted to stay close to my mum and neighbours, so we bought this new apartment with credit. Our apartment is safe, but when I asked the municipality why we couldn't keep our buildings, do you know what they say? They say they would demolish everything to keep the buildings in one style. It's a curse. We've all gotten sick because of this. We've become intolerant, more aggressive—there's no peace in our family. I fight with my husband more often because we both became neurotic, and we're on the verge of divorce. This Project didn't just affect our home: it destroyed our mental stability. We can't tolerate it any longer* (interview, 1 March 2019).

In contrast, owners of extremely old and evacuated buildings face a different situation, with their disappointment and impatience leading to psychological and emotional instability. Hülya, in her sixties, owned a flat damaged in the earthquake in 1999. Declared unsafe, it was evacuated, and she has lived with her daughter ever since. Initially, she believed the Risk Zone Urban Renewal Project would help her return to her neighbourhood, but the planning process dragged on. When I spoke with her, the Project was still awaiting a decision by the Council of State. Hülya described feeling ‘most vulnerable’, having lost the only flat she and her late husband owned and been forced to move in with her daughter because she could not afford to rent alone. What she expected to be temporary turned into full dependence, which she saw as a burden: ‘I'd be homeless without my daughter’. Meanwhile, the evacuated but undemolished buildings around them remained unsafe, yet residents were denied permission to act on their own. ‘We asked to demolish the building ourselves, but the municipality refused. They talk about earthquakes, but we're left waiting endlessly, still paying property taxes’. Hülya felt that years of uncertainty and forced displacement produced more vulnerability than anything else. ‘Sometimes I wish we hadn't been displaced at all—at least we wouldn't feel as vulnerable as we do now’ (interview, 27 February 2019).

Another woman from the same building experienced similar emotional distress and described how the project caused her to lose her social circle:

*I became the scapegoat because I initiated the safety tests for our building after the 1999 earthquake. When it was deemed unsafe, the municipality displaced us. All my neighbours scattered to different parts of the city, and I had to move to a remote area because I couldn't afford rent in a central neighbourhood. Now, my neighbours blame me for everything that happened. It's been years, but I'm still treated like a traitor. All I wanted to know was if our building was safe. This project has turned us into enemies. I've lost all my neighbours and friends—they all blame me now* (interview, 1 March 2019).

Such examples of sociocultural loss and psychological hardship are numerous. Whether people live in newly built homes or older and unsafe buildings, the mental and emotional toll is similar for large segments of the population. These experiences reflect the growing frustration and uncertainty among many residents of the Risk Zone. The extended delays, coupled with the lack of clear communication from the authorities, have exacerbated feelings of helplessness and vulnerability. Residents like Hülya, who had initially hoped that the Project would provide security and a return to normalcy, now confront emotional and financial burdens because of waiting indefinitely, unsure of when—or if—solutions will materialise. This prolonged uncertainty, compounded by the inability to take action on their own, has not only disrupted their lives but also deepened their vulnerability, both socially and economically. Below, I present other cases that highlight different aspects of vulnerability.

### 7.2 Commercial zones and business owners: emergence of future insecurities

In addition to social and mental vulnerabilities, the most frequently expressed concerns, specific economic challenges have also emerged within the Risk Zone. The situation of tradespeople and shop owners is a prime example. They were particularly worried about how they would sustain their businesses during the demolition and construction phases, which could take years. A shop owner, whose business had been passed down for generations, explained that survival depended on long‐standing customers. ‘If the municipality asks us to leave for two years, we won't survive’, he said, stressing that temporary relocation was not a solution. Two or three years away would mean losing loyal customers and paying rent for a new place. ‘I own my shop—why should I risk everything and pay rent elsewhere?’, he asked. On enquiring whether the municipality would cover such rent, the answer was vague: it might be possible, depending on the decision of the Ministry of Environment, Urbanisation and Climate Change. For the shop owner nothing was clear, and everything about the future was uncertain (interview, 11 February 2018).

Furthermore, some shop owners and manufacturers faced the threat of permanent displacement, particularly those categorised as ‘nonfunctional’ and ‘unfitting’ by the Project. A report by Eskişehir Metropolitan Municipality and İTÜ (2017, p. 22) noted that ‘certain areas used for manufacturing and wholesale trade are not suitable for the urban characteristics of Eskişehir's Risk Zone, including storage facilities, printing houses, packaging workshops, repair shops, and other small‐scale production facilities. The presence of these businesses negatively impacts the ambience of the zone’.

Here, the shop owners' fear of displacement was not about the collapse of a building, but about the collapse of a livelihood and a multigenerational social network. While official definitions cast vulnerability as a physical risk to be managed through temporary or permanent evacuation, for shopkeepers it means the prospect of economic dispossession, loss of community trust, and a kind of occupational homelessness that the policy not only failed to prevent but also actively produced.

A real‐estate appraiser from the Project's consultancy company indicated that these shops were expected to be closed by the time the initiative commenced (interview, 23 June 2016). This situation generated fear, anxiety, and anger among both shop owners and renters. Ahmet, a workshop owner in his late seventies who produces small pieces of furniture and offers painting and sanding services, expressed his deep concerns about the future. He knows the mayor of Eskişehir from childhood, as they grew up in the same neighbourhood, and he voted for him before the Project was proposed. Ahmet is extremely worried about what lies ahead:

*I went to the municipality several times to speak with the mayor, but they wouldn't let me in. Why? Because he's a big man now. We used to be neighbourhood friends, and now he's inaccessible. There are rumours that production here [in the workshop] will cease after the Project. We used to be Turkey's top furniture producers, distributing to the entire country, but now we've lost that status to İnegöl. The Project aims to eliminate the last remaining workshops, and I believe the mayor should be protecting us, as we add value to the city. And it's not only us: there will be other shops, like the edge‐tool makers, the print workshops. They say we don't fit here. What a statement! They see us as pollution, a piece of dirt. This isn't just about me: I have workers who depend on this shop. The economic crisis is worsening, and unemployment is rising. What will happen to these people if we lose our business?* (interview, 22 March 2018).

Another shop owner, Osman, also in his seventies, expressed similar worries about the Project's impact on local businesses. He noted that plans to remove hardware shops raise serious questions about where owners will relocate and how they will sustain themselves. He emphasised that no solutions had been proposed by the authorities, leaving shopkeepers to navigate these uncertainties on their own. ‘They just tell us to figure it out ourselves’ (interview, 24 March 2018).

Osman's statement highlights the lack of viable policy solutions. When I enquired about the situation of owners of ‘unfitting’ businesses, many of whom have worked in the neighbourhood their entire life, a municipal officer responded that nothing could be done for them: ‘They'll probably have to move elsewhere because these types of businesses are no longer suitable here. They've lost their functionality in this zone’ (interview, 24 June 2016). These examples illustrate that, unlike the residents of the Risk Zone, shop owners face additional challenges that have not been adequately addressed by the Project's planners and policymakers.

### 7.3 Gender‐ and age‐dependent concerns

The Project's extended planning process has caused further frustration for women and elderly people. During this time, women raised gender‐related concerns. At a knitting and chatting gathering of ladies from a Crimean Tatar cultural association, the undemocratic structure of the Project and its misleading claims were criticised. One woman voiced her frustration with local authorities, asking: ‘Where is the social democratic municipality? What about their promises?’. She noted that while officials claimed that they would not deprive residents, the reality felt very different: ‘We are more deprived now. Is this democracy?’. She explained that years of waiting had eroded trust and patience, leaving her and others simply wanting to be left alone (interview, 11 September 2018). Another woman recalled that whereas their gatherings used to focus on everyday topics such as children and shopping, they were now dominated by discussions about the Project and its potential consequences, particularly the fear of displacement. She emphasised that the community faces problems it cannot resolve; most conversations circulated around rumours and inconsistent information, highlighting a sense of neglect by the municipality.

Women have been particularly concerned about everyday issues caused by the Project, such as household maintenance, which is often seen as their responsibility. While such issues may seem minor as compared to the risks of dispossession, eviction, and mental vulnerability, they add extra burdens that become increasingly difficult to bear over time. Many women have avoided maintenance to prevent unnecessary expense, having heard that the Project could start soon. Hediye, a housewife living with her retired husband, described her frustration with ongoing Project delays, noting that maintaining the home has become difficult. She explained that she often refrains from making repairs, such as painting walls or replacing a boiler, because the municipality repeatedly emphasises that the Project is imminent, and all plans are pending at the Ministry of Environment, Urbanisation and Climate Change. These postponements have forced her to live with broken and deteriorating household items for years, which she finds exhausting. She underlined that while such repairs may seem minor to planners or municipal officials, they represent a significant financial cost for retired residents.

Another matter raised was the ‘social decay’ of the neighbourhoods and issues related to caregiving, particularly among younger women with children. Sinem, from the northern residential part of the Risk Zone, stated:

*Look at the condition of the neighbourhood. On the one hand, there are several unfinished constructions and street repairs. On the other, these dilapidated areas attract people who don't fit our traditional family structure. It's getting dirtier and more threatening to our safety and values. I'm uncomfortable letting my kids play outside anymore. I constantly worry about their safety, even fearing they might be kidnapped. Kids don't want to stay indoors all day; they need to play and expend their energy. That's why I have to take them to other parts of the city or shopping malls to find safe play areas* (interview, 25 September 2018).

In addition to these gender‐related concerns, older residents and pensioners were primarily worried about upcoming payments for the Project. Municipal officers repeatedly stated that their aim was not to ask for large payments from property owners. For instance:

*Residents will need to make small payments for a new flat, viewed as a repair cost for their homes. The upper payment limit was initially set at 50,000 lira [about EUR 15,000 in 2016], but it appears that İTÜ struggled to calculate this accurately and may now require higher payments. We asked İTÜ to work harder on this issue* (interview, 16 June 2016).

Pensioners have expressed significant worries about these payments. İlhan, in his seventies, explained that his pension alone is insufficient to cover living expenses, which forces him to work as a taxi driver at night. He and his wife had saved for years to purchase their flat, but with the prospect of receiving a new one that requires additional payments, even relatively modest amounts of money, such as TRY 10,000, represent a significant burden. He stressed that the municipality appears unaware of retirees' living conditions and needs to take these circumstances into account.

When I asked municipal officers what would happen if residents couldn't pay, they mentioned an option to move into smaller flats. Many locals responded that they couldn't afford the TRY 50,000 payment. Some said they might ask their children for help. When I enquired as to whether they would consider moving into a smaller apartment, pensioners and single individuals said that they would have to think about it. Asiye, a pensioner in her seventies, pointed out that they ‘have to keep a roof over their heads' (interview, 11 March 2016).

Ayşe's story exemplifies the struggles faced by the elderly poor. A widow aged 80‐plus years, she has lived in the same stove‐heated, single‐storey unit for more than 50 years. After her husband passed away several years ago, her children moved to different cities, and she has refused to enter a communal nursing facility because she wants to die in her own home. Living on just TRY 400 a month, she buys a loaf of bread every other day and a 12‐litre water dispenser every 10 days. Her neighbours typically bring her these necessities and prepare her meals. She asked me to convey her plea to the municipality about the potential demolition of her home:

*Daughter, please tell them not to demolish my house. I cannot move elsewhere; this is my home. Where can I go at my age? What can I do? I spend my days here, and sometimes my neighbours visit to bring dinner and heat my stove in winter. May God bless them. Everyone's home is their most beautiful place, isn't it? You may not understand now, but everyone wants to die in their own home. So please convince them not to demolish our houses. They need to think of us; they will grow old one day too. May God bless you* (interview, 29 August 2018).

As all the examples reveal, the transformation of urban areas into a Risk Zone has complex social, economic, and emotional consequences for residents, shop owners, and business operators. The Risk Zone Urban Renewal Project, which claims to mitigate potential disaster risks, inadvertently creates new vulnerabilities, from economic precarity to mental health struggles. Residents of both old, structurally unsafe buildings and newly constructed homes face uncertainty, property value losses, and displacement. The government's rigid approach to demolishing all buildings, regardless of their condition, has fuelled a sense of injustice among property owners, particularly those who invested in newer constructions designed to meet safety standards.

The Project's impacts extend beyond individual households. Local businesses, particularly those deemed incompatible with the area's future development plans, face permanent displacement, risking the livelihoods of owners and employees. Additionally, the slow and opaque decision‐making process erodes trust in local authorities and leaves many people feeling abandoned. Gender‐specific concerns, especially those related to household maintenance and social reproduction, add another layer of complexity, particularly for the women who often bear the brunt of these responsibilities.

## 8 CONCLUSION

This research shows that vulnerability is not only revealed by disaster but produced through its anticipation. By distinguishing between the commodification and the reproduction of vulnerability, the findings shed light on two distinct yet interlinked dynamics of disaster governance. On the one hand, vulnerability is abstracted and instrumentalised to legitimise large‐scale urban interventions—what I have termed its commodification. On the other hand, the very projects designed to reduce risk contribute to new forms of social, psychological, political, and legal precarity—reproducing vulnerability among the populations they claim to protect.

Although grounded in the context of Eskişehir, the dynamics identified here reflect broader patterns in disaster governance that extend beyond this specific city. Anticipatory urbanism, with its forward‐looking yet often technocratic planning logics, combined with narrowly defined framings of safety, increasingly shape how states and municipalities intervene in the built environment. These interventions frequently prioritise infrastructural or market‐centred solutions over the lived experiences of local communities, contributing to the erosion of participatory mechanisms and the marginalisation of local knowledge. Similarly, the commodification of vulnerability—treating risk and precarity as instruments to justify large‐scale projects or facilitate economic interests—has become a recognisable feature of both urban and rural contexts globally. Yet, the ways in which these processes unfold are not uniform: they are deeply conditioned by political structures, economic imperatives, social hierarchies, and historical legacies in each setting. It is also important to note that vulnerability is not inevitably reproduced everywhere; the presence of robust legal protections, meaningful avenues for community engagement, and equitable distribution of resources can mediate the effects of anticipatory governance and even prevent harm from being redistributed to already marginalised populations. These variations underscore the need to analyse disaster governance comparatively, highlighting potential pathways for reform that can reduce risk and safeguard social justice.

Given these insights, this research points to concrete measures for more equitable disaster governance. Definitions of vulnerability should extend beyond structural risk assessments to include social, economic, and emotional dimensions, particularly as articulated by affected populations themselves. Public communication must shift from alarmist or paternalistic messaging towards dialogue, participatory planning, and knowledge coproduction. Legal frameworks, including Law No. 6306, should be reassessed to ensure protective interventions do not override rights to housing, representation, or continuity of place. Institutional reforms could include strengthened oversight mechanisms, mandatory impact assessments for marginalised populations, and avenues for meaningful community input throughout the planning process.

Ultimately, this case underscores the enduring value of ethnographic research in disaster studies. It reveals the limits of conventional risk management, affirms the knowledge and agency of affected communities, and offers a roadmap for disaster governance that is both socially grounded and attentive to historical inequalities. By centring the experiences of those most affected, we can move towards policies that not only prevent a disaster itself, but also the reproduction of vulnerability.

## Data Availability

The data that support the findings of this study are available from the corresponding author upon reasonable request.

## Endnotes

^1^
The Department of Architecture at İTÜ was responsible for the planning of the Risk Zone Urban Renewal Project.

^2^
For a detailed analysis of the previous urban renewal projects and lawfare between the Eskişehir Metropolitan Municipality and the local population, see Civelek (2023).
